# Time correlation of success recanalization for endovascular recanalization of medically refractory non-acute intracranial arterial occlusions

**DOI:** 10.3389/fsurg.2022.1074514

**Published:** 2023-01-06

**Authors:** Zhiyong Ji, Yeping Ling, Pingbo Chen, Yuxiao Meng, Shancai Xu, Pei Wu, Chunlei Wang, Tatiana Ilyasova, Bowen Sun, Huaizhang Shi

**Affiliations:** ^1^ Department of Neurosurgery, The First Affiliated Hospital of Harbin Medical University, Harbin, China; ^2^ Department of Internal Diseases, Bashkir State Medical University, Ufa, Russian Federation

**Keywords:** ischemic stroke, atherosclerosis, endovascular therapy, intracranial large vessel occlusion, nonacute occlusion

## Abstract

**Background and purpose:**

The management of patients with symptomatic non-acute atherosclerotic intracranial artery occlusion (sNAA-ICAO), which is a special subset with high morbidity and a high probability of recurrent serious ischemic events despite standard medical therapy, has been clinically challenging. A number of small-sample clinical studies have discussed endovascular recanalization for sNAA-ICAO and the lack of a uniform standard of operation time. The purpose of this study was to investigate the time correlation of successful recanalization.

**Methods:**

From January 2013 to August 2021, 69 consecutive patients who underwent endovascular recanalization for sNAA-ICAO were analyzed retrospectively in the First Affiliated Hospital of Harbin Medical University. The technical success rate, periprocedural complications, and rate of TIA/ischemic stroke during follow-up were evaluated.

**Results:**

The overall technical success rate was 73.91% (51/69), and the rate of perioperative complications was 37.68% (26/69). The percentage of patients with perioperative symptoms was 27.53% (19/69). The rate of serious symptomatic perioperative complications was 8.70% (6/69). After adjusting for age, sex, and BMI, the effect of the time from the last symptom to operation on successful recanalization was 0.42 (IQR, 0.20, 0.88, *P* = 0.021), before the inflection point (51 days).

**Conclusions:**

Endovascular recanalization for sNAA-ICAO is technically feasible in reasonably selected patients. The perioperative safety is within the acceptable range. Before 51 days, the last symptoms to operation time, for every 10 days of delay, the probability of successful recanalization is reduced by 57%.

## Introduction

Intracranial artery occlusive disease is an important cause of ischemic stroke (1, 2). In the therapeutic window, previous randomized studies have shown the overwhelming benefit of endovascular therapy for patients with acute intracranial artery occlusion (3–7). Symptomatic non-acute intracranial artery occlusion beyond 24 h from the onset is a particular type of intracranial artery occlusive disease. A number of these patients will continue to be at higher risk for subsequent stroke despite aggressive medical therapy, especially those with hemodynamic damage (8, 9). Recently, some small-sample clinical studies have reported that endovascular recanalization is feasible for symptomatic non-acute atherosclerotic intracranial artery occlusion (sNAA-ICAO). However, the optimal treatment and best therapeutic window for these patients remain unclear, leading to heterogeneity of the outcomes and perioperative complications between these studies (10–15). In the present study, we report the time correlation of success recanalization for endovascular recanalization of medically refractory nonacute intracranial arterial occlusions.

## Methods

### Study population

Cases were identified through the hospital information system acquired endovascular databases of the First Affiliated Hospital of Harbin Medical University. From January 2013 to August 2021, 69 consecutive patients with sNAA-ICAO treated with endovascular recanalization were reviewed. Fifty of them were men, with an average age of 58.74 ± 9.75 years. Based on the initial symptoms (transient ischemic attack (TIA) or stroke beyond 24 h), patients were diagnosed with intracranial artery occlusion by magnetic resonance angiography (MRA) or computed tomography angiography (CTA), and then these patients were treated with optimal medical therapy (dual antiplatelet therapy plus statin under management of risk factors). The reasons for not receiving intravenous thrombolysis or thrombectomy included interhospital transfer beyond 24 h from the onset, rapid relief of symptoms, or mild [National Institutes of Health Stroke Scale (NIHSS) score < 4] initial symptoms. During clinical observation and follow up, endovascular treatment was considered when patients still experienced recurrent TIA or stroke related to the occluded artery. Recurrent TIA was defined as a clinical syndrome of acute focal loss of brain function related to the occluded artery, with symptoms resolving within 24 h. Recurrent stroke was defined as any new focal neurological deficit associated with the occluded artery, with a sudden onset, with symptoms lasting at least 24 h. This study was approved by the institutional review board of Harbin Medical University. The data were anonymous, and the requirement for patient-informed consent for the review of patient records and images was waived.

### Inclusion criteria

The inclusion criteria are as follows: (1) non-acute intracranial artery occlusion, diagnosed by CTA or MRA and confirmed by digital subtraction angiography (DSA); (2) time of the last symptom to recanalization over 24 h; (3) recurrent TIA or new stroke related to the occluded intracranial artery despite aggressive medical therapy and risk control; (4) more than one risk factor able to cause atherosclerosis, including hypertension, diabetes mellitus, hyperlipidemia, coronary artery disease, and cigarette smoking; (5) intracranial artery, including intracranial internal carotid artery (Ic-ICA), intracranial vertebral artery (Ic-VA), basilar artery (BA), and middle cerebral artery (MCA); and (6) a preoperative modified Rankin scale (mRS) score of 3 or less.

### Exclusion criteria

The exclusion criteria are as follows: (1) clinical, laboratory, or imaging findings suspicious for nonatherosclerotic lesions such as vasculitis, moyamoya syndrome, or arterial dissection; (2) data on clinical and imaging costs; (3) concomitant ≥50% stenosis of the proximal intracranial artery or other intracranial arteries; and (4) a life expectancy of <1 year due to other medical conditions.

### Endovascular recanalization procedure

All procedures were performed under general anesthesia. After placement of the sheath introducer, heparin was administered intravenously to keep the activated clotting time between 200 and 300 s. Half of the dose is given 1 h later; if the procedure lasts more than 2 h, a quarter of the initial dose would be given every hour thereafter. A 6 French (6F) guiding catheter was advanced into the proximal of the occluded intracranial artery. A microwire (Synchro, Stryker Neurovascular, Fremont, CA; Transend EX 014/205 soft tip, Boston Scientific Corp., Natick, MA) was used in combination with a microcatheter (Echelon-10, ev3 Neuro-vascular, Irvine, CA; Excelsior SL-10, Stryker Neurovascular, Fremont, CA; Headway, MicroVention, Fremont, CA), which were carefully passed through the occluded segment. If, after repeated attempts, the microwire and microcatheter could not pass through the occluded segment and enter the true distal lumen, the procedure was stopped. If the microcatheter was successfully guided through the occluded segment, then a microcatheter injection would be used to confirm the position distal to the occluded segment in the true distal lumen. Subsequently, an exchange microwire (Transend ES 014/300 Floppy, Boston Scientific Corp., Natick, MA) was placed in the appropriate anchoring position, and the microcatheter was removed. The size of the balloon was selected according to the diameter of the occlusion lumen; usually 80% of the diameter of the vessel. If many perforators were present adjacent to the vessel, a balloon with a size of 60% of the vessel diameter was used. An angioplasty balloon (Gateway, Stryker Neurovascular, Fremont, CA; SFX Soloflex, Sinomed, CHN) was advanced over the exchange microwire across the lesion. If the balloon could not pass through the occluded segment or the many perforators in the occluded segment with higher risks, the procedure was stopped. If the occluded segment was passed, balloon dilation was performed on the occluded segment, and if the occlusion length exceeded 15 mm, it was expanded sequentially from the distal to the proximal segment of the lesion. If the residual stenosis was still severe after the first balloon dilation, a larger balloon was used for redilation. Based on measurement of the proximal and distal diameters, as well as the length of the occluded segment after balloon dilation, a self-expandable stent (Wingspan stent, Stryker Neurovascular, Fremont, CA; Neuroform EZ, Stryker Neurovascular, Fremont, CA; or Enterprise, Codman & Shurtleff, Raynham, MA) was introduced and deployed according to the manufacturers’ instructions. Postoperative angiography was performed to confirm the patency. Successful revascularization is defined as antegrade flow with a modified thrombolysis in the cerebral infarction (mTICI), with a grade of 3 and residual stenosis ≤50% (16). Brain CT was performed immediately after the operation to rule out intracranial hemorrhage. All patients were typically monitored in a neurocritical care unit for 24 h after the procedure, with a systolic blood pressure target of <120 mmHg to reduce the risk for reperfusion hemorrhage. A serious symptomatic perioperative complication is defined as an increase of 4 or more points in the NIHSS score.

### Data collection

Cases were identified through the hospital information system acquired endovascular databases of the First Affiliated Hospital of Harbin Medical University. The following baseline and treatment variables were collected: demographic, clinical, procedural, and imaging data. Two independent neurosurgeons carried out all image assessments, and discrepancies were resolved by consensus.

### Statistical analysis

The baseline data, perioperative results, and clinical and imaging data for the successful reperfusion group and the unsuccessful reperfusion group were compared. All normally distributed continuous and quantitative variables were expressed as mean ± SD, non-normally distributed variables were expressed as median and interquantile range (IQR), and categorical variables were expressed as proportions. Comparisons between groups were performed using the Kruskal–Wallis test, Fisher's precision probability test for continuous variables, and a *χ*^2^ test for categorical variables. According to the differences between groups, parameters with *P* < 0.5 were selected for univariate analysis. Threshold effect analysis was performed on the parameters, with high correlation, to determine the relationship between exposure and outcome variables. If the threshold effect analysis determined an inflection point, smooth curve fitting was performed to obtain the corresponding effect value. Differences were considered statistically significant when *P* ≤ 0.05. All statistical analyses were performed with R (http://www.R-project.org) and EmpowerStats (www.empowerstats.com; X&Y Solutions, Inc., Boston, MA) software.

## Results

### Baseline characteristics

All 69 patients underwent endovascular recanalization for sNAA-ICAO. The median time from initial radiological diagnosis to endovascular treatment was 26.0 days (IQR 9.0–47.0), and the median time from last symptom onset to endovascular treatment was 40.0 days (IQR 29.0–64.0). Most of the occluded sites were posterior circulation large vessel occlusions; Ic-VA accounted for 43.48% of the total. The time from the last symptom to recanalization was longer in the unsuccessful recanalization group than in the successful recanalization group. Still, there was no significant difference in the patients’ demographic characteristics, risk factors, or clinical characteristics between the successful and unsuccessful reperfusion groups. Detailed baseline information is provided in Table 1.

**Table 1:** Baseline characteristics.

	Overall (*n* = 69)	Successful reperfusion (*n* = 51)	Unsuccessful reperfusion (*n* = 18)	*P*-value
Mean age, years	58.74 ± 9.75	59.31 ± 9.31	57.11 ± 11.02	0.331
Men, no. (%)	50 (72.46%)	35 (68.63%)	15 (83.33%)	0.230
BMI	25.18 ± 2.97	24.80 ± 3.15	26.21 ± 2.16	0.077
Preoperative mRS score, median (IQR)	1 (0–1)	1 (0–2)	0 (0–1)	0.116
Preoperative NIHSS score, median (IQR)	0 (0–2)	1 (0–3.5)	0 (0–1)	0.060
Occlusion site, no. (%)				0.290
BA	17 (24.64%)	15 (29.41%)	2 (11.11%)	
Ic-VA	30 (43.48%)	19 (37.25%)	11 (61.11%)	
Ic-ICA	10 (14.49%)	8 (15.69%)	2 (11.11%)	
MCA	12 (17.39%)	9 (17.65%)	3 (16.67%)	
Risk factors, no. (%)				
Hypertension	50 (72.46%)	38 (74.51%)	12 (66.67%)	0.522
Diabetes mellitus	17 (24.64%)	13 (25.49%)	4 (22.22%)	0.782
Cardiac disease	15 (21.74%)	11 (21.57%)	4 (22.22%)	0.954
Hyperlipidemia	3 (4.35%)	3 (5.88%)	0 (0.00%)	0.293
Hyperhomocysteinemia	13 (28.26%)	8 (23.53%)	5 (41.67%)	0.230
Smoking				0.437
Have not quit	16 (23.19%)	10 (19.61%)	6 (33.33%)	
Have quitted	7 (10.14%)	5 (9.80%)	2 (11.11%)	
Qualifying events, no. (%)				0.393
Recurrent TIA	23 (33.33%)	15 (29.41%)	8 (44.44%)	
Recurrent stroke	44 (63.77%)	34 (66.67%)	10 (55.56%)	
Serum total cholesterol	3.98 ± 1.03	3.99 ± 0.93	3.95 ± 1.29	0.602
Triglyceride	1.83 ± 0.94	1.94 ± 1.04	1.56 ± 0.54	0.186
Low-density lipoprotein	2.42 ± 0.79	2.45 ± 0.75	2.34 ± 0.90	0.390
First symptom to recanalization (days), median (IQR)	62 (36–100)	57 (35–119)	63 (40–77.75)	0.779
Last symptom to recanalization (days), median (IQR)	40 (29–64)	39 (27.5–62.5)	47.5 (39–69.25)	0.098
Initial radiological diagnosis to recanalization (days), median (IQR)	26 (9–47)	23 (9–46)	28.5 (10.5–46.5)	0.661

mRS, modified Rankin scale; IQR, interquantile range; NIHSS, National Institutes of Health Stroke Scale.

### Perioperative outcomes

The overall technical success rate was 73.91% (51/69). The perioperative complication rate was 37.68% (26/69). Table 2 shows the operative parameters and perioperative complications for the patients. There was a significant difference in the operation time between the two groups. The operation was finished for the patients in the successful recanalization group in 90 (IQR, 72.5–120) min. For the unsuccessful recanalization group, the operation was finished in 75 (IQR, 48.75–90) min. In the successful recanalization group, five patients (9.8%) did not require stent release because the lumen stenosis was significantly improved and maintained after balloon dilation during the operation, or there were many perforators in the occluded segment, and ischemic stroke was caused by stent release. The stent was not released due to risk. In the nonsuccessful recanalization group, the occluded segment could not be passed to terminate the operation in 13 cases, the microguide wire and microcatheter passed the occluded segment vessels and reached the true lumen in five cases, in four cases it was difficult to reach the distal end with the balloon and guide wire, and in the other cases, after balloon dilatation, considering the risk of perforating vessel occlusion, no further treatment was performed and only positive blood flow was restored; however, the stenosis rate was more than 50%.

**Table 2:** Perioperative outcomes.

	Overall (*n* = 69)	Successful reperfusion (*n* = 51)	Unsuccessful reperfusion (*n* = 18)	*P*-value
Operation time (min), median (IQR)	90 (60–105)	90 (72.5–120)	75 (48.75–90)	0.029*
Occlusion length, mm	23.28 ± 12.33	22.23 ± 12.26	26.27 ± 12.36	0.209
Endovascular treatment				
Microwire passage	56 (81.16%)	51 (100%)	5 (27.78%)	−
Microcatheter passage	56 (81.16%)	51 (100%)	5 (27.78%)	−
Balloon angioplasty	52 (75.36%)	51 (100%)	1 (5.56%)	−
Stenting	46 (66.67%)	46 (90.20%)	0 (0%)	−
Intraoperative complications				
Dissection	6 (8.70%)	2 (3.92%)	4 (22.22%)	0.018*
Perforation	1 (1.45%)	0 (0.00%)	1 (5.56%)	0.090
In-stent thrombosis	1 (1.45%)	1 (1.96%)	0 (0.00%)	0.550
Periprocedural complications	26 (37.68%)	22 (43.14%)	4 (22.22%)	0.115
Intracerebral hemorrhage				0.472
Intraparenchymal hemorrhage	4 (5.80%)	4 (7.84%)	0 (0.00%)	
Subarachnoid hemorrhage	7 (10.14%)	5 (9.80%)	2 (11.11%)	
Symptomatic intracranial hemorrhage	4 (5.80%)	3 (5.88%)	1 (5.56%)	0.959
In-stent re-occlusion	3 (4.35%)	3 (5.88%)	−	−
Perioperative transient ischemic attack or ischemic stroke				0.460
Transient ischemic attack	4 (5.80%)	4 (7.84%)	0 (0.00%)	
Ischemic stroke	12 (17.39%)	9 (17.65%)	3 (16.67%)	
Other complications	3 (4.35%)	3 (5.88%)	0 (0.00%)	0.293
NIHSS at discharge (median) (IQR)	0 (0–3)	0 (0–3)	0 (0–1)	0.296
mRS at discharge (median) (IQR)	0 (0–2)	0 (0–2)	1 (0–1)	0.729

*P < 0.05.

A total of eight patients (11.59%) exhibited intraoperative complications, most of which were arterial dissection; two patients showed dissection during operation, and the dissection disappeared after stent release. In addition, four patients' operations were terminated due to arterial dissection that could not break through to the true lumen, and DSA did not show further dissection expansion after surgery. In one patient, when the Traxcess microwire and the Headway 17 microcatheter tried to pass through the occlusion site, microcatheter ultraselective angiography confirmed extravasation of the contrast agent, the Target 2*6 mm, 1.5*3 mm, 1.5*4 mm, and 1*3 mm coils were released, and the operation was terminated after no contrast medium extravasation was confirmed by angiography. Moreover, in one patient, after the stent was released, the contrast agent circulated slowly and remained in the left posterior cerebral artery, which was considered to be caused by plaque shedding and blockage. Left intracranial carotid artery (ICA) angiography showed that the posterior communicating artery (PComA) opened to compensate, and further treatment was not continued.

A total of 26 (37.68%) perioperative complications occurred; ischemic complications were higher than hemorrhagic complications, and cerebral parenchymal hemorrhage occurred in the successful recanalization group. The percentage of patients with perioperative symptoms was 27.53% (19/69). The rate of serious symptomatic perioperative complications was 8.70% (6/69).

There were only four cases of symptomatic intracranial hemorrhage. One patient with left intracranial ICA occlusion was successfully recanalized. After going to the toilet on the third day after the operation, the patient developed sudden inarticulate speech and could not move the right limb. Emergency CT showed a hemorrhage in the left temporal lobe. Decompressive craniectomy and evacuation of intracerebral hematoma were performed. The mRS score was 5 after discharge.

One patient who underwent interventional recanalization of the left MCA occlusion vomited once after returning to the neurosurgical intensive care unit 30 min after the operation. Physical examination showed that the patient experienced tingling pain and stretched limbs and was unable to open their eyes or make a sound. CT showed that a left basal ganglia cerebral hemorrhage broke into the ventricle and a subarachnoid hemorrhage (SAH) followed; emergency hematoma trepanation, drainage, and decompressive craniectomy were performed and after discharge the mRS score was 5.

Another patient underwent interventional recanalization of the left MCA occlusion and drowsiness occurred 7 h after the operation. The muscle strength was grade 1, the right lower extremity muscle strength was grade 2, and the GCS score was 11. CT showed cerebral hemorrhage, subarachnoid hemorrhage, and multiple cerebral infarctions in the left basal ganglia. After conservative treatment, the patient was discharged from the hospital with clear speech and a deviated tongue. The proximal area of the right upper limb was grade 3, the right distal end of the upper limb was grade 1, the remaining limbs were grade 5, and the discharge mRS score was 3.

The last patient was recanalized after BA occlusion. Intraoperative balloon dilation and re-examination showed that the blood vessel returned to normal blood flow. However, there were still apparent residual stenosis and dissection changes, and the distal blood flow imaging was poor. Postoperative routine re-examination head CT showed a slightly higher density in the front of the brainstem and left frontal lobe, and symptoms of diplopia and vertigo occurred. The discharge mRS score was 1.

Among the three cases of perioperative re-occlusion, one case was BA occlusion interventional recanalization. On the seventh day after the operation, the right limb was numb, weak, and slightly clumsy. After a few minutes of observation, the patient's limb movement disorder worsened, and physical examination showed that he was clear with clumsy speech, exhibiting a shallow right nasolabial fold, a right limb muscle strength of grade 3, and a positive pathological reflex. After CT re-examination, consciousness disturbance, the rigidity of limbs, and positive pathological signs of both lower extremities were found. Emergency DSA examination showed basilar artery stent thrombosis and BA occlusion; 700,000 units of urokinase was pumped into the artery, and the patient woke up immediately after surgery. Physical examination showed that the right limb muscle strength was grade 4, the left limb muscle strength was grade 5, and the pathological reflex of the right lower limb was suspicious. On the first day after thrombolysis, the muscle strength of the right limb was slightly weak. At the time of discharge, the speech was clumsy, the right limb was slightly clumsy, the muscle strength was grade 4, the pathological reflex was negative, and the discharge mRS score was 2.

In one case of a patient with BA occlusion who received recanalization, the patient went into a coma 2 h after the operation, with bilateral mydriasis, the disappearance of light reflex, and high limb muscle tension. Emergency intra-arterial thrombolysis was performed. DSA showed thrombosis in the BA stent. In total, 800,000 units of urokinase was slowly pumped into the artery, and re-examination showed that BA was unobstructed; however, the patient's prognosis was poor. One patient had the same symptoms as the third patient, with symptomatic craniocerebral hemorrhage. Postoperative MRA showed that the left middle cerebral artery was not clearly displayed, and MCA occlusion was considered. The patient was discharged from the hospital with a clear voice, and the tongue held to the right. The proximal upper extremity was grade 3, the distal right upper extremity was grade 1, and the remaining limbs were grade 5. The discharge mRS score was 4.

During the 90-day follow-up, only three patients had died. All of them had severe clinical complications. Two patients had perioperative intracranial hemorrhage and one patient had perioperative re-occlusion.

### Univariate analysis

According to the previous comparison between groups and previous literature, the time from the first symptom to operation, time from the last symptom to operation, time from confirmed occlusion to operation, and occlusion length were included in univariate analysis and adjusted, respectively. Table 3 lists the corresponding OR values and *P* values.

**Table 3:** Univariate analysis of successful recanalization.

	Crude OR (95% CI) *P*-value	Adjusted 1 OR (95% CI) *P*-value	Adjusted 2 OR (95% CI) *P*-value
First symptom to recanalization (days)	1.00 (1.00, 1.00) 0.853	1.00 (1.00, 1.00) 0.964	1.00 (1.00, 1.00) 0.963
Last symptom to recanalization (days)	1.00 (1.00, 1.01) 0.830	0.99 (0.98, 1.01) 0.236	0.99 (0.98, 1.01) 0.225
Initial radiological diagnosis to recanalization (days)	1.00 (0.99, 1.01) 0.672	1.00 (0.98, 1.01) 0.736	1.00 (0.98, 1.01) 0.725
Occlusion length, mm	0.97 (0.93, 1.02) 0.233	0.98 (0.94, 1.03) 0.427	0.98 (0.93, 1.03) 0.387

Crude, no adjustment; Adjusted 1, adjusted for age, gender, and BMI; Adjusted 2, adjusted for age, gender, BMI, and anterior and posterior circulation vessels.

### Threshold effect analysis

Univariate analysis did not show a clear univariate effect on successful recanalization. However, after adjustment, the effect size of the time from the last symptom to operation on successful recanalization changed significantly, and the two factors of time from the last symptom to operation and the occlusion length were adjusted for age, sex, BMI, and a *P* value < 0.5. The time from the last symptom to operation and the occlusion length were selected for threshold effect analysis, as shown in Table 4.

**Table 4:** Threshold effect analysis of related factors of successful recanalization.

	Last symptom to operation (days)	Occlusion length, mm
Model I (linear fitting)	0.99 (0.98, 1.01)	0.98 (0.94, 1.03)
Model II (curve fitting)		
Inflection point (K)	45	9.63
<K	0.91 (0.84, 0.99)*	1.57 (0.94, 2.63)
>K	1.00 (0.98, 1.02)	0.96 (0.91, 1.01)
Efficiency shifting between <K and >K	1.10 (1.00, 1.20)*	0.61 (0.36, 1.05)
Likelihood ratio (LR)	0.023	0.082

*P < 0.05.

In the threshold effect analysis, the log-likelihood ratio test of the time from the last symptom to operation was 0.023, indicating an inflection point in the effect of time from the last symptom to operation on successful recanalization.

**Figure 1:**
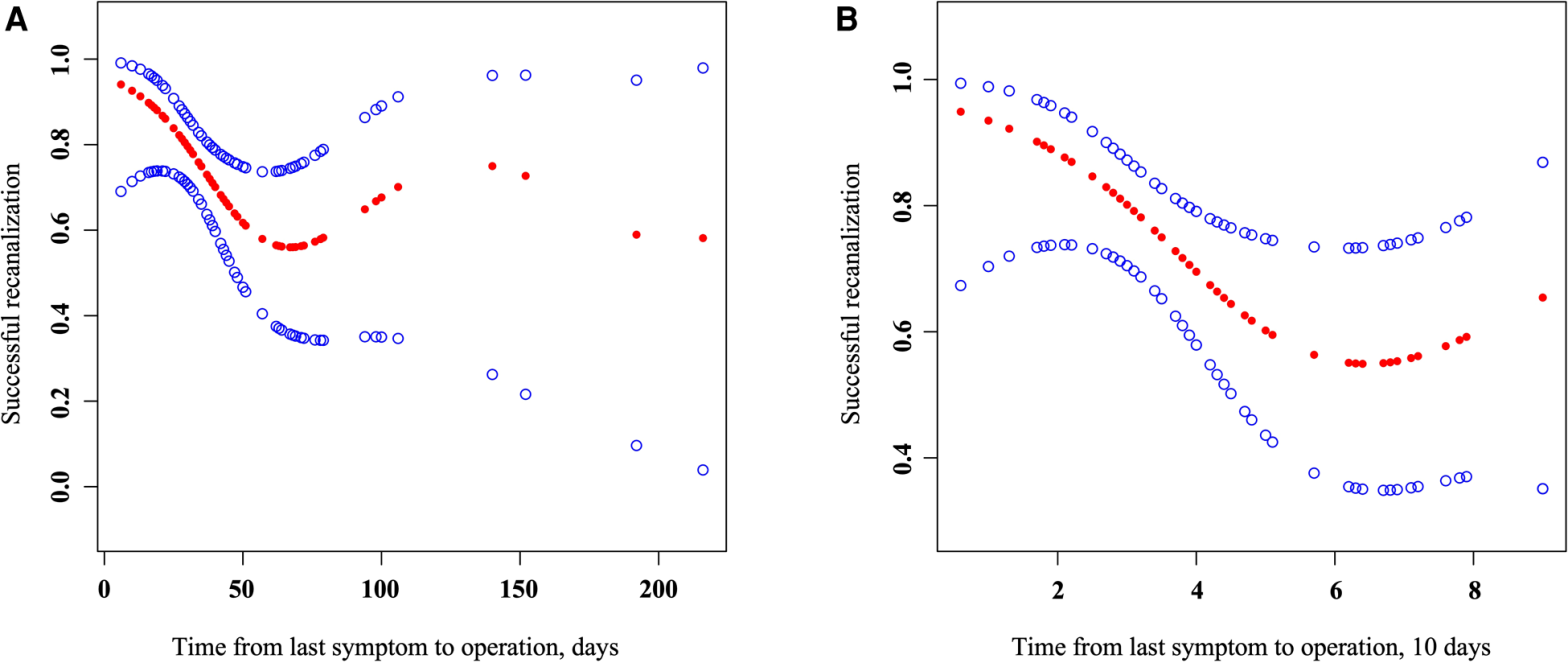
Association of the time from the last symptom to operation with successful recanalization. Smooth curve fitting (left) of the effect of the time from the last symptom to operation (days) on successful recanalization, adjusted for age, gender, and BMI; smooth curve fitting (right) of the eddect of the time from the last symptom to operation (10 days) on successful recanalization; parameters larger than 100 days were refitted in 100 days, adjusted for age, gender, and BMI.

Smooth curve fitting was performed on the effect of time from the last symptom to operation on successful recanalization. As shown in Figure 1A, the curve fitting between the time from the last symptom to the operation and successful recanalization became gradually discrete beyond 90 days, and the amount of data was small. After adjusting the original data, the curve fitting shown in Figure 1B was obtained.

As shown in the Table 5, After adjusting for age, gender, and BMI, after another threshold effect analysis, every additional 10 days before the 51-day inflection point decreased the probability of successful recanalization by 57%, with an effect size *β* of 0.43 (0.21, 0.88) and *P* = 0.021. After further adjustment for anterior and posterior circulation vessels, the effect was still significant: *β* = 0.39 (0.18, 0.84) and *P* = 0.016.

**Table 5:** Threshold effect analysis of the time from the last symptom to operation on successful recanalization.

Last symptom to recanalization (10 days)	Adjusted 1 *β* (95% CI) *P*-value	Adjusted 2 *β* (95% CI) *P*-value
<5.1	0.43 (0.21, 0.88) 0.021*	0.39 (0.18, 0.84) 0.016*
>5.1	1.26 (0.81, 1.95) 0.302	1.30 (0.83, 2.05) 0.255
Efficiency shifting between <5.1 and >5.1	2.95 (1.05, 8.28) 0.040*	3.32 (1.11, 9.89) 0.032*
Likelihood ratio (LR)	0.027	0.019

Adjusted 1, adjusted for age, gender, and BMI; Adjusted 2, adjusted for age, gender, BMI, and anterior and posterior circulation vessels. *P < 0.05.

## Discussion

This study shows that interventional recanalization therapy is feasible and effective for screened sNAA-ICAO patients. The recanalization rate of this study technique reached 73.91%, which is an acceptable range compared with recanalization rates of 53.1%–95.5% in published articles in the past 3 years (16–20); the Ic-VA occlusion recanalization rate was 63.33%, which was the lowest. In addition, this study retrospectively analyzed the patient data from 2013 to 2021. The time span was large. In the early days, the center carried out sNAA-ICAO interventional recanalization less frequently; with the increase of the learning time curve and the extension of case screening time, the overall recanalization rate became larger. Moreover, with the establishment of the Stroke Center, a large number of patients with acute ischemic stroke (AIS) do not have to undergo nonacute occlusion before opening, and early opening also determines the vascular conditions of patients who can be selected *via* sNAA-ICAO, which leads to a less likely recanalization intervention.

In this study, the perioperative complication rate was 26 (37.68%), including asymptomatic craniocerebral hemorrhage. After excluding this cohort of patients, the percentage of patients with perioperative symptoms was 27.53% (19/69). The rate of serious symptomatic perioperative complications was 8.70% (6/69), including symptomatic craniocerebral hemorrhage and major stroke. Presently, the perioperative complication rate reported by other centers ranges from 6.25% to 31.8%, and is mainly around 20% (10–13, 16–18, 21–23). The incidence of complications in this study is slightly higher than that in other studies, partly due to the lack of early HR-MRI study of the blood vessel wall, blood vessel plaque, and prediction of the direction of occluded blood vessels, which often increases the corresponding surgical risk and perioperative period. Another cause of the occurrence of complications is hemorrhagic stroke, which mainly leads to anterior circulation interventional recanalization, especially MCA interventional recanalization, and may be due to the lack of primary and secondary compensation such as the circle of Willis, AComA, and PComA. The load on the distal vascular bed is lower, and there is a higher risk of bleeding after successful recanalization. Therefore, preoperative screening and postoperative blood pressure control still require further research. In addition, two of the hemorrhagic strokes in this study were delayed, suggesting that sudden perioperative blood pressure fluctuations may be related to hemorrhage. The onset of ischemic stroke and TIA is one of the most common perioperative complications. Many of them are acute lacunar infarctions found during postoperative DWI, which is considered to be related to interventional operations. However, most of the symptoms are mild and can be significantly improved by active medical treatment and the improvement of circulatory symptoms. Two patients developed symptoms after successful recanalization. Repeat angiography found that the deep perforating branch of BA and the lenticulostriate artery disappeared. Successful technical recanalization restored normal blood flow and met the vessel's diameter requirements, yet the risk of occlusion of adjacent vessels remained. It needs to be carefully considered by the surgeon during the operation whether successful recanalization is beneficial to the patient and whether restoring normal blood flow alone can benefit further research. In addition, some patients presented with ischemic stroke caused by cerebral vasospasm and complicated by hemorrhagic stroke.

Among the factors influencing successful recanalization in this study, only the time from the last symptom to operation was significant. The time from the last symptom to operation in this study was 40 (IQR, 29–64) days; much longer than the time from the last symptom to operation studied by the Feng team in the past 3 years (16–18, 24). In total, 75% of the IQR was concentrated before 21 days. This may be due to the low overall recanalization rate and the appearance of a U-shaped curve in the impact on successful recanalization. This study showed that after adjusting for age, gender, and BMI, every additional 10 days before the 51-day inflection point decreased the probability of successful recanalization by 57%. It was also confirmed that thrombus fibrosis was organized, and the microguidewire's passage rate after calcified plaques was significantly reduced, resulting in a decrease in the rate of successful recanalization in the previous research. For obviously organized lumens, it is often considered that the occlusion time is longer, and the possibility of successful recanalization is lower (12, 25, 26). Nevertheless, if the lumen is present and collapsed the occlusion time may be shorter. This was also confirmed in a recent high-resolution study, with HR-MRI showing higher successful recanalization of occlusions in the residual lumen (27). Therefore, due to early intervention in previous studies, the mid- and long-term effects of the time from the last symptom to operation on successful recanalization were often not shown. However, after the inflection point (51 days), the U-shaped curve exhibited a trend of increasing the successful recanalization rate; however, there is no statistical significance. The primary consideration is selection bias. Imaging often prompts interventional recanalization for patients with longer occlusion times. So, after 51 days, there is an overall enlargement of a select few of the overall population.

## Conclusion

Endovascular recanalization for sNAA-ICAO is technically feasible in reasonably selected patients, and perioperative safety is within the acceptable range. Before 51 days, for the last symptoms to operation time, for every 10 days of delay, the probability of successful recanalization is reduced by 57%.

## Data Availability

The raw data supporting the conclusions of this article will be made available by the authors, without undue reservation.
